# Does postoperative exercise after hip arthroplasty improve strength and function? A systematic review and meta-analysis

**DOI:** 10.1186/s12891-026-10107-5

**Published:** 2026-06-19

**Authors:** Zeyu Ye, Sai Yu, Leming Liao, Zebin Zhang, Li Lv

**Affiliations:** 1 Fuyang First People’s Hospital Affiliated to Zhejiang Chinese Medical University (Hangzhou Fuyang First People’s Hospital), No. 300 Zhujian Road, Chunjiang Subdistrict,Fuyang District, Hangzhou City, Zhejiang Province 311400 China; 2 Fuyang campus of Zhejiang Provincial People’s Hospital (The First People’s Hospital of Fuyang), No. 300 Zhujian Road, Chunjiang Subdistrict, Fuyang District, Hangzhou City, 311400 China

**Keywords:** Total hip arthroplasty, Osteoarthritis, Postoperative exercise, Rehabilitation, Meta-analysis, Muscle strength, Harris Hip Score

## Abstract

**Objective:**

To determine the clinical benefits of structured postoperative exercise on muscle strength, patient-reported function, and range of motion after total hip arthroplasty (THA) for osteoarthritis.

**Methods:**

We searched multiple databases for randomized controlled trials and prospective studies comparing structured exercise versus standard care after primary THA. Pooled effects were calculated using standardized mean differences (SMD) or mean differences (MD) with random‑effects models. Subgroup analyses were stratified by follow‑up duration (< 3 months, 3–6 months, > 12 months) and assessed limb (operated vs. healthy).

**Results:**

Twenty‑two studies (1,782 participants) were included. For hip abduction strength, exercise significantly improved short‑term (< 3 months; SMD ‑0.78, 95% CI: ‑1.19 to ‑0.36) and mid‑term (3–6 months; SMD ‑0.69, 95% CI: ‑1.10 to ‑0.29) outcomes, but not long‑term (> 12 months; SMD ‑0.29, 95% CI: ‑0.75 to 0.17). Hip flexion strength showed no significant effect (SMD 0.10, 95% CI: ‑0.27 to 0.47). The Harris Hip Score improved significantly at 2 weeks (MD ‑4.62), 4 weeks (MD ‑7.67), 6 weeks (MD ‑17.83), and 12 weeks (MD ‑3.05), but the 1‑year effect was uncertain (MD ‑17.94, 95% CI: ‑49.13 to 13.25; I²=99.1%). Hip range of motion analysis was inconclusive (I²=94.4%). GRADE certainty was moderate for short‑/mid‑term hip abduction strength and early HHS improvements, and low to very low for other outcomes.

**Conclusion:**

Structured postoperative exercise provides short‑ to mid‑term improvements in hip abduction strength and early patient‑reported function, but long‑term efficacy and effects on hip flexion strength remain unconfirmed.

## Introduction

Total Hip Arthroplasty (THA) is a highly effective intervention for end-stage osteoarthritis, reliably alleviating pain and restoring basic mobility. However, the role of structured postoperative exercise in optimizing functional recovery remains a subject of considerable debate. A substantial body of systematic reviews and meta-analyses has yielded conflicting conclusions, creating uncertainty for clinical practice. Some high-profile reviews have questioned the efficacy of postoperative exercise. Notably, a comprehensive meta-analysis by Saueressig et al., [1] concluded there was low- to moderate-quality evidence that postoperative exercise was not associated with improved self-reported physical function at various time points up to one year. This contrasts with other syntheses focusing on specific outcomes or prehabilitation, which have reported benefits for function, pain, and strength [1–3].

Our review is built upon a broad international evidence base encompassing studies from Europe, Asia, North America, and Australia, ensuring the generalizability of the conclusions. A critical examination of this contradictory literature reveals two significant limitations that may underlie the inconsistent findings. First, many reviews aggregate outcomes broadly, potentially obscuring muscle group-specific effects. For instance, conclusions about “muscle strength” may not distinguish between key functional muscle groups like the hip abductors, essential for gait stability, and the hip flexors. Second, the temporal trajectory of potential benefits is often inadequately characterized, with effects pooled across widely varying follow-up durations from weeks to over a year, masking the dynamic nature of post-THA recovery.

Therefore, to address these evidence gaps and provide a more nuanced understanding, this systematic review and meta-analysis investigates the clinical question: Does structured postoperative exercise provide clinically meaningful benefits after total hip arthroplasty for osteoarthritis? We focus on three critical and distinct outcome domains to move beyond generalized conclusions: (1) Hip abduction strength, a key objective measure of abductor muscle recovery crucial for gait and function; (2) Hip flexion strength, to assess if benefits are specific to certain muscle groups; and (3) the Harris Hip Score, a patient-reported gold standard encompassing pain, function, and range of motion. Furthermore, we analyze these outcomes stratified by postoperative follow-up time to delineate the evolution of any treatment effects. By providing this granular, time-sensitive analysis of discrete outcome measures.We acknowledge that language used in the context of body weight and obesity can carry potential risks of harm, and we have adopted person-first language throughout this manuscript to promote inclusivity and respect for all individuals. this study aims to clarify the specific value and optimal timing of structured exercise in post-THA rehabilitation.

Therefore, to address these evidence gaps and provide a more nuanced understanding, this systematic review and meta-analysis investigates the clinical question: Does structured postoperative exercise provide clinically meaningful benefits after total hip arthroplasty for osteoarthritis? We focus on three critical and distinct outcome domains to move beyond generalized conclusions: (1) Hip abduction strength, a key objective measure of abductor muscle recovery crucial for gait and function; (2) Hip flexion strength, to assess if benefits are specific to certain muscle groups; and (3) the Harris Hip Score, a patient-reported gold standard encompassing pain, function, and range of motion. Furthermore, we analyze these outcomes stratified by postoperative follow-up time to delineate the evolution of any treatment effects. We prespecified the direction of effect modification, expecting that exercise would demonstrate clinically meaningful benefits in short- to mid-term outcomes but that these advantages would attenuate by 12 months post-operatively. By providing this granular, time-sensitive analysis of discrete outcome measures, this study aims to clarify the specific value and optimal timing of structured exercise in post-THA rehabilitation.

## Materials and methods

This systematic review and meta-analysis was conducted in accordance with the Preferred Reporting Items for Systematic Reviews and Meta-Analyses (PRISMA) statement [4].

### Search strategy and selection criteria

A comprehensive literature search was performed in the PubMed, Web of Science, EMBASE, Scopus and ProQuest database from its inception until January 2026. The search strategy was designed to identify studies investigating structured exercise interventions following total hip arthroplasty for osteoarthritis. Key search terms included controlled vocabulary (MeSH terms) and free-text words related to the population (“total hip arthroplasty”, “hip replacement”), the intervention (“exercise”, “rehabilitation”, “training”, “physiotherapy”), and the study designThe detailed search strategy, including the full Boolean logic string, is provided in the Supplementary Appendix.). The complete search strategies for each databaseincluding PubMed, Web of Science, EMBASE, Scopus, and ProQuestare provided in Supplementary Table S1. For PubMed, the full search string was: (“Arthroplasty, Replacement, Hip“[Mesh] OR “total hip arthroplasty“[tiab] OR “total hip replacement“[tiab] OR THA[tiab] OR THR[tiab]) AND (“Exercise Therapy“[Mesh] OR “Rehabilitation“[Mesh] OR exercise*[tiab] OR train*[tiab] OR rehab*[tiab] OR physiotherap*[tiab]) AND (“Osteoarthritis, Hip“[Mesh] OR osteoarthritis[tiab]) AND (random*[tiab] OR controlled[tiab] OR trial[tiab]). For other databases, the search string was adapted to the respective syntax and controlled vocabulary. Detailed adaptations are listed in the supplementary material. The search was restricted to English-language studies on human subjects. The reference lists of identified systematic reviews and included articles were also screened for additional eligible studies.

### Search strategy and selection criteria

A comprehensive literature search was performed in the PubMed, Web of Science, EMBASE, Scopus and ProQuest database from its inception until January 2026. The search strategy was designed to identify studies investigating structured exercise interventions following total hip arthroplasty for osteoarthritis. Key search terms included controlled vocabulary (MeSH terms) and free-text words related to the population (“total hip arthroplasty”, “hip replacement”), the intervention (“exercise”, “rehabilitation”, “training”, “physiotherapy”), and the study design. The following Boolean logic search string was used and adapted as needed:

(“Arthroplasty, Replacement, Hip“[Mesh] OR “total hip arthroplasty“[tiab] OR “total hip replacement“[tiab] OR THA[tiab] OR THR[tiab]) AND (“Exercise Therapy“[Mesh] OR “Rehabilitation“[Mesh] OR exercise*[tiab] OR train*[tiab] OR rehab*[tiab] OR physiotherap*[tiab]) AND (“Osteoarthritis, Hip“[Mesh] OR osteoarthritis[tiab]) AND (random*[tiab] OR controlled[tiab] OR trial[tiab]). The reference lists of identified systematic reviews and included articles were also screened for additional eligible studies. The search was restricted to studies on human subjects. The reference lists of identified systematic reviews and included articles were also screened for additional eligible studies.

### Eligibility criteria

Studies were included if they met the following PICOS criteria: (1) Population: Adult patients (≥ 18 years) undergoing primary total hip arthroplasty for osteoarthritis. (2) Intervention: Any structured, supervised, or prescribed postoperative exercise or rehabilitation program, initiated after surgery. (3) Comparison: Usual care, minimal intervention, or a different (typically less intensive) rehabilitation protocol. (4) Outcomes: At least one of the following: hip abduction strength, hip flexion strength, Harris Hip Score (HHS), or hip joint range of motion (ROM). Other functional and patient-reported outcomes were also recorded. 5)Study Design: Randomized controlled trials (RCTs) and comparative studies (prospective or retrospective).

### Eligibility criteria

Studies were included if they met the following PICOS criteria: (1) Population: Adult patients (≥ 18 years) undergoing primary total hip arthroplasty for osteoarthritis. (2) Intervention: Any structured, supervised, or prescribed postoperative exercise or rehabilitation program, initiated after surgery. (3) Comparison: Usual care, minimal intervention, or a different (typically less intensive) rehabilitation protocol. (4) Outcomes: At least one of the following: hip abduction strength, hip flexion strength, Harris Hip Score (HHS), or hip joint range of motion (ROM). Other functional and patient-reported outcomes were also recorded.5) Study Design: Randomized controlled trials (RCTs) and comparative studies (prospective or retrospective).

### Data extraction and management

Two reviewers independently screened titles, abstracts, and full texts. A standardized data extraction form was piloted on five randomly selected studies and refined accordingly before full extraction. Discrepancies were resolved through discussion or consultation with a third reviewer. From each included study, data were extracted on: study characteristics (author, year, design), participant demographics, details of the intervention and control protocols, follow-up duration, outcome measures, and results. For continuous outcomes, we extracted the mean, standard deviation (SD), and sample size for both the intervention and control groups at baseline and at the specified postoperative follow-up time points. If post-intervention SDs were not available, they were calculated from standard errors, confidence intervals, or p-values where possible.

### Assessment of risk of bias

We conducted a Grading of Recommendations, Assessment, Development and Evaluations (GRADE) assessment to evaluate the certainty of evidence for key outcomes: hip abduction strength, hip flexion strength, Harris Hip Score, and hip range of motion. The GRADE approach considers study design, risk of bias, inconsistency, indirectness, imprecision, and publication bias to rate evidence as high, moderate, low, or very low certainty.

Discrepancies in risk of bias ratings between the two reviewers were resolved through consensus discussion; if consensus could not be reached, a third reviewer was consulted to make the final decision. The methodological quality and risk of bias of the included studies were critically appraised using design-specific tools by two independent reviewers. For RCTs, the revised Cochrane Risk of Bias tool (RoB 2.0) was employed. This tool evaluates bias across five domains: (1) bias arising from the randomization process, (2) bias due to deviations from intended interventions, (3) bias due to missing outcome data, (4) bias in measurement of the outcome, and (5) bias in selection of the reported result. Each domain was judged as leading to a “low risk of bias,” “some concerns,” or “high risk of bias,” culminating in an overall risk-of-bias judgment for each specific outcome. For the included non-randomized studies (e.g., prospective or retrospective cohort studies), the Risk Of Bias In Non-randomized Studies – of Interventions (ROBINS-I) tool was applied [5]. ROBINS-I assesses bias across seven domains: bias due to confounding, bias in selection of participants, bias in classification of interventions, bias due to deviations from intended interventions, bias due to missing data, bias in measurement of outcomes, and bias in selection of the reported result. Similar to RoB 2.0, judgments were made at the domain and overall study level.

### Statistical analysis

All statistical analyses were performed using R (version 4.3.2) with the meta package (version 6.5-0) [6]. The primary analysis compared the change from baseline to follow-up between intervention and control groups. For outcomes measured on the same scale (e.g., Harris Hip Score), we calculated the mean difference (MD) with 95% confidence interval (CI); for outcomes with different measurement methods or units (e.g., muscle strength measured by different dynamometers), we used the standardized mean difference (SMD) with Hedges’ g (correcting for small sample bias).

#### Directionality convention

A negative pooled effect size (MD or SMD) favours the intervention group, and a positive value favours the control group. Accordingly, in all forest plots, the left side of the null line represents the direction favouring the intervention.

The 95% CIs for pooled effect sizes were derived using a normal approximation based on standard errors from the inverse‑variance method and the REML estimate of between‑study variance (τ²). For analyses including more than two studies with τ² > 0, we applied the Hartung‑Knapp variance adjustment. Confidence intervals for pooled MD and SMD used the Wald‑type approximation (standard error of the pooled estimate × z = 1.96 for 95% level). A random‑effects model was applied for all primary analyses using the inverse‑variance method, anticipating substantial clinical and methodological heterogeneity.

Within‑study correlations from multiple time points or effect sizes in the same cohort were addressed by separate subgroup analyses stratified by follow‑up time. Each time point was treated as an independent unit within its respective subgroup (e.g., < 3 months, 3–6 months, > 12 months), consistent with the random‑effects assumption of independence between subgroups. This approach does not explicitly model within‑study correlation but minimizes its impact by preventing pooling of effects from the same study across different time windows, thereby preserving clinical distinctness of recovery phases.

Between‑study heterogeneity was quantified using τ² (estimated via restricted maximum likelihood, REML) and the I² statistic. To mitigate potential underestimation of variance and inflated type I error rates in meta‑analyses with < 10 studies, we used the Hartung‑Knapp‑Sidik‑Jonkman method for all primary analyses, providing more conservative interval estimates. The Q‑profile method calculated the CI for τ².

We pre‑specified the direction of effect modification by follow‑up time, hypothesizing larger intervention effects in the short term (< 3 months) than mid‑term (3–6 months) or long term (> 12 months), with diminishing strength effects over time. Multiple time points from the same study were included in different temporal subgroups; we treated each time point as independent because data came from separate participant cohorts at each follow‑up (no participant contributed to more than one time point within the same subgroup). This avoids double‑counting and respects clinical distinctness of recovery phases. Multilevel models or meta‑regression could account for within‑study correlations but were precluded due to the limited number of studies per subgroup and the non‑overlapping nature of the data points.

Pre‑specified subgroup analyses investigated heterogeneity: for hip abduction strength and HHS, we stratified by follow‑up duration (e.g., < 3 months, 3–6 months, > 12 months, and specific weeks where data allowed). For hip abduction strength with short‑term follow‑up (≤ 3 months), we additionally stratified by assessed limb (operated vs. healthy). Differences between subgroups were tested using a mixed‑effects model.

Publication bias was not formally assessed using funnel plots or Egger’s test for most outcomes because each analysis included fewer than 10 studies, below the recommended threshold for reliable asymmetry tests; we acknowledge its potential impact. To quantify the credibility of subgroup findings, we applied the ICEMAN tool, which evaluates eight criteria (e.g., direction of effect modification, prior evidence plausibility, within‑ and across‑study evidence, statistical significance) to generate a credibility rating (high, moderate, low, or very low). Two reviewers independently assessed each subgroup analysis and resolved disagreements by consensus.

We considered multilevel models or meta‑regression to account for dependency among multiple effect sizes from the same study, but their implementation requires a substantial number of comparisons and well‑characterized between‑study covariates not fully met in our dataset. We therefore opted for a subgroup approach stratified by clinically meaningful follow‑up windows, which sufficiently addresses the nested structure of time points while maintaining interpretability. Results were visually summarized using forest plots generated with the forest.meta() function from the meta package.

## Results

**Fig. 1:**
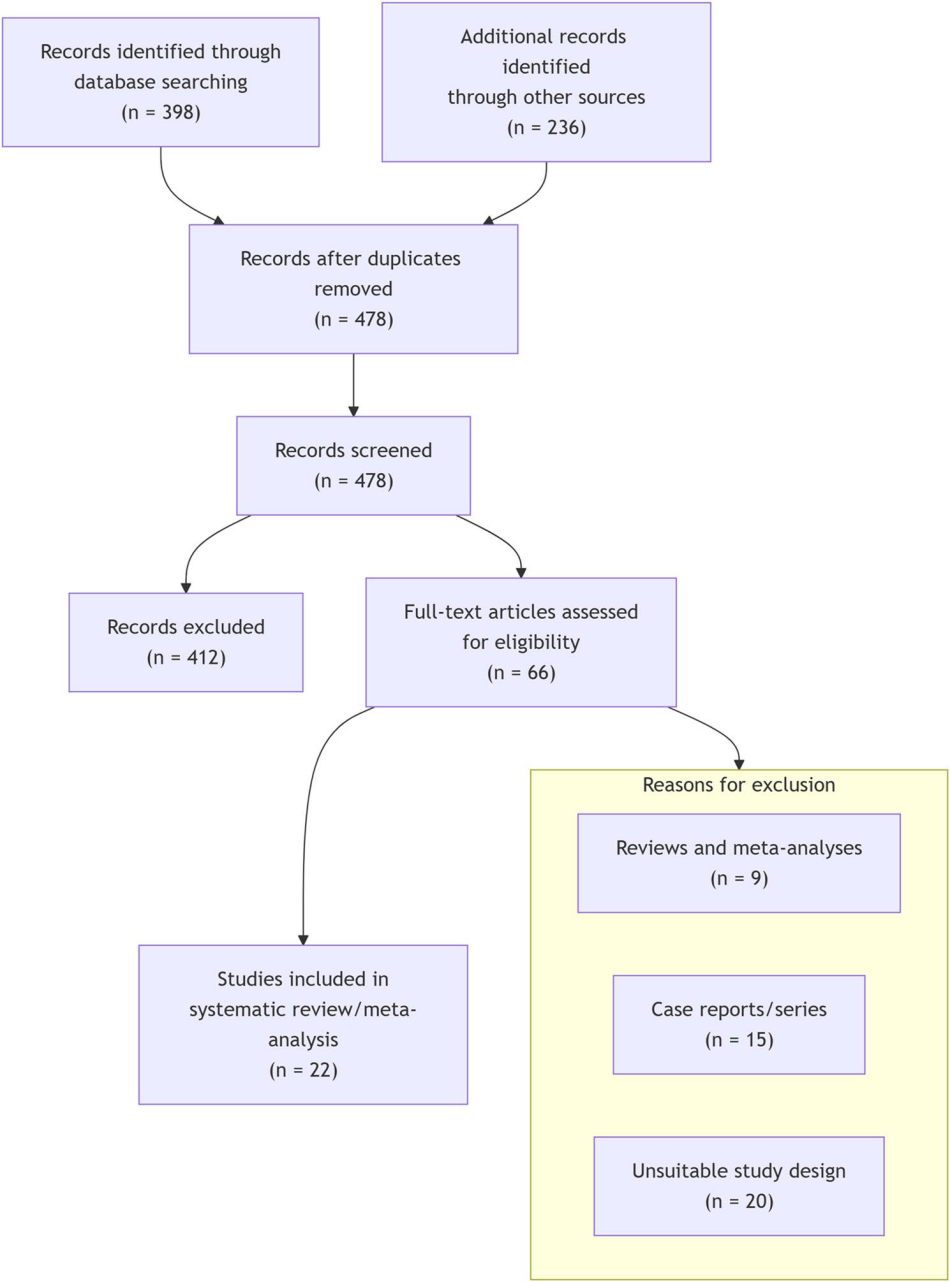
PRISMA flow diagram of study selection for the systematic review and meta-analysis

Of the 634 records identified from database searches, 22 studies ultimately met the pre-defined inclusion criteria and were included in this systematic review and meta-analysis (Fig. 1).

A total of 634 records were initially identified through systematic searches of electronic databases and other sources. After the removal of 156 duplicates, 478 unique records were screened based on title and abstract. Following this initial screening, 412 records were excluded, leaving 66 full-text articles assessed for eligibility In the context of our eligibility criteria, which restricted inclusion to randomized controlled trials (RCTs) and prospective comparative studies, the 20 studies categorized as having an unsuitable study design included retrospective cohort studies (k = 8), case-control studies (k = 4), cross-sectional studies (k = 3), single-arm interventional trials without a control group (k = 3), and non-English language publications with insufficient extractable data (k = 2). Of these, 44 articles were excluded due to being reviews or meta-analyses (*n* = 9), case reports/series (*n* = 15), or having an unsuitable study design (*n* = 20). The flow diagram was generated following the PRISMA 2009 template. Ultimately, 22 studies met the pre-defined inclusion criteria and were incorporated into the systematic review and meta-analysis (Fig. 1).

### Characteristics of included studies

This systematic review and meta-analysis ultimately included 22 independent studies published between 2003 and 2025. The majority of the included studies were randomized controlled trials (RCTs, k = 17), complemented by five retrospective or prospective cohort studies. Geographically, the studies originated from diverse regions including Europe, Asia, North America, and Australia. All studies exclusively enrolled patients undergoing primary total hip arthroplasty for osteoarthritis. The median proportion of female participants across studies was approximately 58%. The structured postoperative exercise interventions were heterogeneous, encompassing modalities such as supervised progressive resistance training, home-based exercise programs, gait/balance training, and combined exercise-nutrition approaches; these were compared against various control conditions, primarily usual care or less intensive rehabilitation protocols. The primary outcomes of interest across the studies varied but consistently included measures of muscle strength, functional performance, and patient-reported outcomes A detailed summary of the characteristics of each included study, including the specific exercise protocols and control conditions, is provided in Supplementary Table 1. The follow-up duration for outcome assessment exhibited considerable range, from immediate postoperative periods (e.g., 1–2 weeks) up to 12 months post-surgery, enabling the planned subgroup analyses by time (Table 1).

**Table 1:** Characteristics of Included Studies

Study	Country	Study period	Study Design	Sample size	Disease	Intervention drugs/methods	Primary endpoint	Secondary endpoint	Inclusion Criteria	Exclusion Criteria	Follow-up period
Gilbey et al. 2003 [7]	Australia	1997–1998	RCT	76	Osteoarthritis	Customized perioperative exercise program; Routine in-hospital physical therapy only	N/A	N/A	Diagnosis of hip arthritis. chronic pain/disability unresponsive to conservative treatment. stable health fit for anesthesia	History of hip infection. significant neuromuscular disease. hip malignancy. poor general health. revision or bilateral hip surgery	24 weeks postoperatively
Unlu et al. 2007 [8]	Turkey	2006–2007	RCT	26	Osteoarthritis	Home exercise program; Supervised in-hospital exercise; Walking only	Max isometric hip abduction torque	Gait speed. Cadence	THA patients 12–24 months post-surgery	Neuro. cognitive. metabolic diseases. early complications. revisions. other joint problems	6 weeks
Husby et al. 2009 [9]	Norway	N/A	RCT. ID 00638417	24	Osteoarthritis	Maximal strength training + conventional rehab; Conventional rehab only	Muscle strength (1RM leg press and abduction)	Work efficiency. gait patterns. quality of life	Age < 70 years. primary OA for THA. ASA score PI	Muscular/skeletal. heart/lung diseases. diabetes	5 weeks postoperatively
Husby et al. 2010 [10]	Norway	N/A	RCT (00638417)	24	Osteoarthritis	Max strength training + conv rehab; conv rehab only	Work efficiency	Muscle strength. gait patterns. VO2max. quality of life	Age â‰¤70. primary OA. ASA class 1	Musculoskeletal diseases. cardiac/pulmonary diseases. diabetes	6 and 12 months
Tsukagoshi et al. 2012 [11]	Japan	Jun 2004-Aug 2007	Retrospective cohort study	30	Hip osteoarthritis	Stepping exercises plus conventional physical therapy; Conventional physical therapy only	Lower limb muscle strength and walking speed	N/A	Women undergoing primary unilateral THA for hip osteoarthritis	Revision THA. bone graft or femoral osteotomy. incomplete testing	6 wks
Heiberg et al. 2012 [12]	Norway	Oct 2008-Mar 2010	RCT. NCT00808483	68	Osteoarthritis of hip. THA	Walking skill training program (12 sessions); No physiotherapy	6-minute walk test (6MWT)	Stair climbing test. figure-of-eight test. IMF. hip ROM. HHS. self-efficacy. HOOS	OA hip diagnosis. residence near hospital	OA knee/contralateral hip restricting walking. neurologic disease. dementia. heart disease. drug abuse. inadequate Norwegian language	12 months
Mikkelsen et al. 2012 [13]	Denmark	2008–2009	RCT	46	Osteoarthritis	IG: intensified home-based exercises with rubber band resistance; CG: standard exercises without external resistance	Maximal gait speed improvement	Hip abduction strength. one-legged stance. EQ-5D. WOMAC. patient satisfaction	Patients scheduled for primary unilateral THR for osteoarthritis in fast-track programme	Discharge to rehabilitation unit or cognitive/language problems	12 weeks
Beaupre et al. 2014 [14]	Canada	N/A	RCT	21	Osteoarthritis	comprehensive postoperative exercise program; usual postoperative care	N/A	N/A	Age < 65 years. primary unilateral THA with Hardinge approach. living in metropolitan area	Primary diagnosis of developmental dysplasia of the hip	6 weeks. 4 months. 12 months postoperatively
Mikkelsen et al. 2014 [15]	Denmark	Sep 2010-Nov 2012	RCT. NCT01214954	73	Osteoarthritis	supervised PRT 2 days/week + home exercise 5 days/week; home exercise 7 days/week	change in leg extension power at 10 weeks	isometric hip strength. sit-to-stand. stair climb. 20 m walk. HOOS	Primary unilateral THR for OA. HOOS ADL â‰¤67. age > 18. local residence. willing to train	Resurfacing hip. BMI > 35. planned rehab. contralateral THR within 6 months. language/mental/physical barriers	10 weeks
Umpierres et al. 2014 [16]	Brazil	Jul 2009-Oct 2011	RCT NCT01491048	106	Osteoarthritis with THA	THAP group: verbal instructions and exercise demonstrations; THAPCP group: verbal instructions and demonstrations with physiotherapist-guided daily exercise practice	N/A	N/A	Patients with hip OA undergoing THA	Refused participation. lived in another city. cognitive disorders. THA for hip fracture	15 days
Rampazo-Lacativa & D’Elboux. 2015 [17]	Brazil	N/A	RCT NCT01622465	N/A	Hip osteoarthritis with total hip arthroplasty	Group I: cycle ergometer and conventional exercises; Group II: conventional exercises alone	Function evaluated using HHS and SPPB	HRQOL measured using SF-36 and WOMAC	Older patients (â‰¥60 years) with primary unilateral THA for hip osteoarthritis	Hip fracture. postoperative complications. revision arthroplasty. neuromuscular disease. unable to attend sessions. refusal to participate	6 months
Nankaku et al. 2016 [18]	Japan	N/A	RCT	28	hip osteoarthritis with THA	hip external rotator exercise; conventional rehab	N/A	N/A	primary THA for unilateral hip OA. 4-week follow-up. asymptomatic contralateral side	neuromuscular disease. cardiovascular problems. revision THA. rheumatoid arthritis. other musculoskeletal diseases	4 weeks
Mikkelsen et al. 2017 [19]	Denmark	2015–2016	RCT; secondary analysis of intervention group from RCT NCT01214954	37	Osteoarthritis	Supervised progressive resistance training (2 days/week) plus home-based exercise (5 days/week); Unsupervised home-based exercise program	Training load and hip pain response to training	Training attendance and adverse events	Primary unilateral THA for osteoarthritis. preoperative HOOS ADL â‰¤67. age > 18. residence within 30 km of hospital	BMI > 35 kg/m2. preplanned postoperative rehab. inability to speak/read Danish. mental/physical conditions impeding intervention	10 weeks
Mitrovic et al. 2017 [20]	Serbia	January 2013 to June 2015	RCT ISRCTN73197506	70	Hip osteoarthritis	supplementary arm and upper body exercises; standard rehabilitation programme	Harris Hip Score	Hand grip strength. SF-36 Health Survey	Older than 60 years with end-stage primary hip osteoarthritis undergoing primary unilateral total hip replacement	Postoperative complications. cognitive impairment. congenital hip dislocation. bilateral hip disease or inflammatory arthritis. significant neuromuscular disease. lower extremity fractures or paralysis	12 weeks
Winther et al. 2018 [21]	Norway	Aug 2015-Feb 2016	RCT NCT02498093	60	Osteoarthritis	MST (leg press and abduction at 85–90% 1RM. 3x/week for 3 months); CP (exercises with low/no external loads)	Abduction and leg press strength at 3 months	Pain. 6MWT. HHS. HOOS-PS	Primary OA patients scheduled for elective THA	Severe contralateral hip OA. prior THA not recovered. communication issues. rehab discharge. conditions affecting training/testing	3.6.12 months postop
Beck et al. 2019 [22]	Germany	Jan 2015â€“Jul 2017	RCT NCT03584451	160	Osteoarthritis of hip with THR	Hip rehab sports therapy once weekly; No sports therapy	Change in hip muscle strength at 12 months	Changes in strength at 6 months. postural stability. cardiopulmonary endurance. patient-reported outcomes	General medical eligibility for hip rehab sports therapy. stable implant. age â‰¥18 years. written consent	Acute or chronic diseases. severe pain in affected hip	12 months
Mikami et al. 2019 [23]	Japan	Jan 2017-May 2018	å›žé¡¾æ€§é˜Ÿåˆ—ç ”ç©¶	44	Hip osteoarthritis with THA	Conventional physical therapy; Conventional physical therapy plus anti-gravity treadmill training and low-frequency electrical stimulation	N/A	N/A	Patients undergoing THA for hip osteoarthritis	Patients with walking difficulty. non-Japanese speakers. inability to consent due to psychiatric symptoms. or inability to complete evaluations	2 weeks
Ninomiya et al. 2023 [24]	Japan	March 2021 to October 2021	RCT (UMIN 000042964)	58	Hip osteoarthritis	Exercise therapy plus nutritional supplementation (protein and vitamin D); Exercise therapy alone	Hip abductor and knee extensor muscle strengths	Grip strength. quadriceps muscle thickness. intramuscular adipose tissue. functional performance (TUG-t. HHS). hip pain VAS. QOL (JHEQ). laboratory data (ALB. Hb. CRP)	Female aged 65–80 years with prefrailty/frailty undergoing primary THA for unilateral OA without severe ROM restriction	Serious medical/orthopaedic conditions. previous hip surgery. food allergies. neurological/muscular disorders. dementia. schizophrenia	12 weeks
Tian et al. 2022 [25]	China	Jan 2018 to Jan 2021	Retrospective cohort study	539	Hip osteoarthritis or femoral head necrosis	Routine gait rehabilitation; Bed exercise plus routine gait	N/A	N/A	Unilateral femoral head necrosis ARCO 3–4 or osteoarthritis KL 3–4. ageâ‰¥60. healthy and stable for anesthesia	Poor general condition. non-weight-bearing postop. complex THA requiring bone grafting/screws. inability to walk independently. hip infection or malignancy. neuromuscular diseases	17 weeks
Chen et al. 2024 [26]	China	2021–2022	RCT. ChiCTR-2,300,072,553	90	Hip osteoarthritis	Preoperative combined with postoperative progressive resistance training; Preoperative or postoperative progressive resistance training alone	Isokinetic muscle strength (HMIT-NBM and SLAR)	Gait parameters. balance. and hip function (HHS)	Patients with unilateral or bilateral hip osteoarthritis scheduled for unilateral primary THA	Contraindications for exercise. recent surgery. severe comorbidities	12 months
Zhang et al., 2025 [27]	China	Dec 2021-Dec 2023	Retrospective cohort study	97	Hip pathology requiring HA (mixed etiologies)	routine nursing and rehabilitation exercises; stepped rehabilitation training plus standard care	Hip joint function and bone metabolism markers	Quality of life and postoperative complications	Radiologically confirmed hip pathology with HA indication, normal preoperative muscle strength, ASA II-IV, unilateral primary HA, complete data	Prior hip surgery, cognitive/psychiatric disorders, acute infection, other fractures or joint diseases, severe organ dysfunction, bone tumor, coagulation dysfunction, hip neuropathy/suppuration, pre-surgery non-independence	4 weeks
Queen et al., 2015 [28]	USA	N/A	Prospective cohort study	42	Hip osteoarthritis	N/A	Limb asymmetries in lower extremity mechanics during stair climbing	Patient-reported outcomes (Harris Hip Score, SF-12)	Age > 35 years, scheduled for primary THA	Lower extremity surgery in past 5 years, neurological disorders, pain in other joints	1 year

### Risk of bias assessment

According to the GRADE assessment (Table 2), the certainty of evidence for hip abduction strength was moderate for short- and mid-term follow-up (downgraded due to risk of bias), and low for long-term follow-up (downgraded for risk of bias and imprecision). For hip flexion strength, the evidence certainty was moderate (downgraded due to imprecision). For Harris Hip Score, evidence certainty ranged from moderate (early time points) to very low (1-year follow-up, downgraded due to risk of bias, inconsistency, and imprecision). For hip range of motion, evidence certainty was very low (downgraded due to risk of bias and inconsistency).

**Table 2:** GRADE evidence profile for primary outcomes

Outcome	Follow-up	No. of studies (participants)	Risk of bias	Inconsistency	Indirectness	Imprecision	Publication bias	Overall certainty
Hip abduction strength (SMD)								
	Short-term (< 3 months)	9 (287)	Serious (−1)	Not serious	Not serious	Not serious	Not rated (*n* < 10)	Moderate
	Mid-term (3–6 months)	4 (108)	Serious (−1)	Not serious	Not serious	Not serious	Not rated (*n* < 10)	Moderate
	Long-term (> 12 months)	3 (89)	Serious (−1)	Not serious	Not serious	Serious (−1)	Not rated (*n* < 10)	Low
Harris Hip Score (MD)								
	Early (1–2 weeks)	2 (78)	Serious (−1)	Not serious	Not serious	Not serious	Not rated (*n* < 10)	Moderate
	Mid (6–8 weeks)	2 (86)	Serious (−1)	Serious (−1)	Not serious	Not serious	Not rated (*n* < 10)	Low
	Late (1 year)	2 (72)	Serious (−1)	Very serious (−2)	Not serious	Serious (−1)	Not rated (*n* < 10)	Very low
Hip range of motion (MD)	4 weeks	2 (125)	Serious (−1)	Very serious (−2)	Not serious	Serious (−1)	Not rated (*n* < 10)	Very low

All downgrading decisions are based on data explicitly reported in the Results section. Publication bias was not rated due to fewer than 10 studies per analysis. The “Importance” column is omitted because it is not consistently defined in the manuscript

**Fig. 2:**
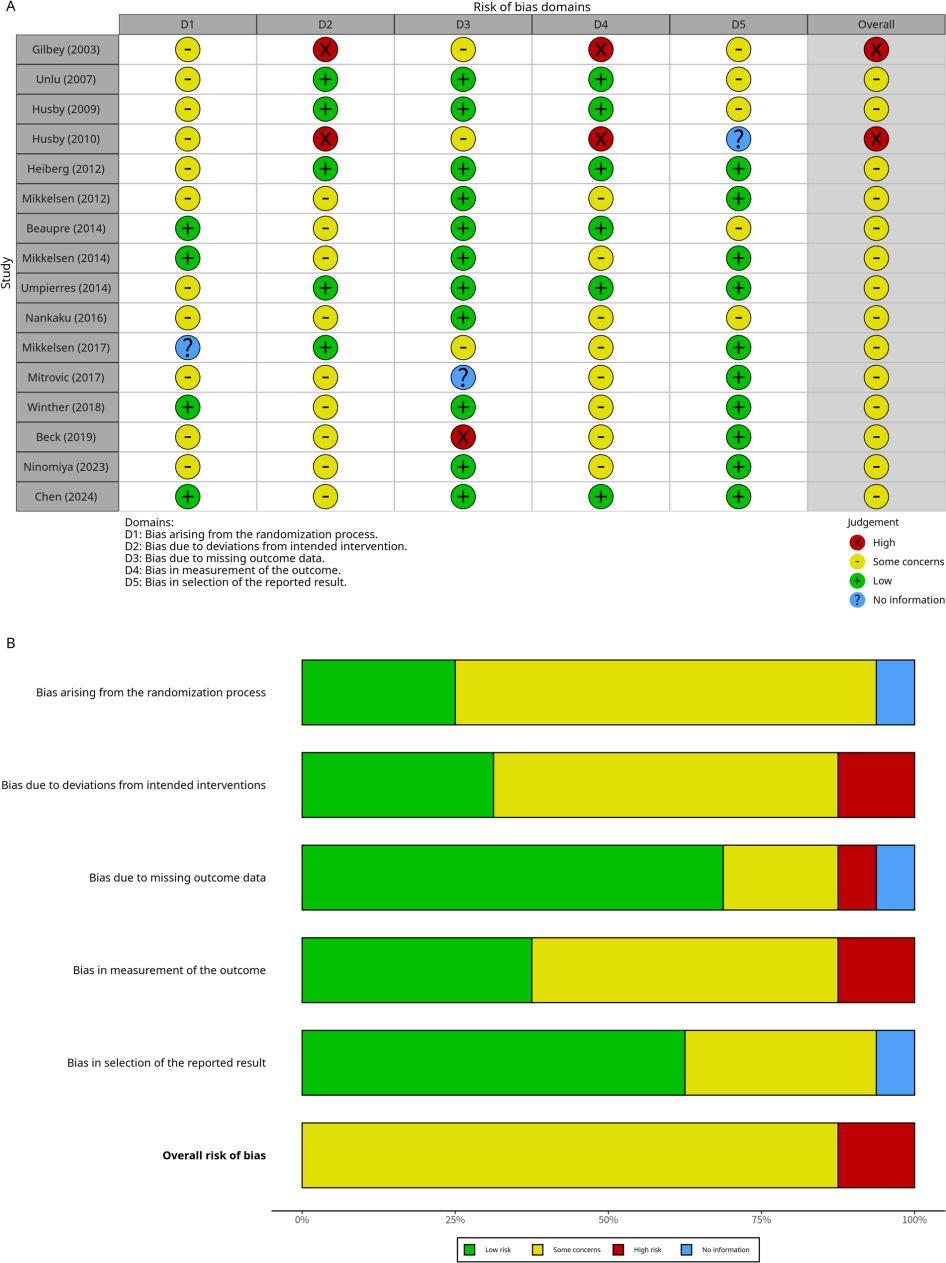
Risk of Bias Assessment Using the RoB 2 Tool for Randomized Trials. **A** Traffic Light Plot of Domain-Level Risk-of-Bias Judgements. This panel displays the risk-of-bias judgements (low risk: green; some concerns: yellow; high risk: red) for each domain across individual included studies. **B** Summary Bar Plot of the Distribution of Risk-of-Bias Judgements. This panel shows the proportion (or weighted proportion) of studies judged as low risk, some concerns, or high risk within each bias domain, including overall risk of bias

**Fig. 3:**
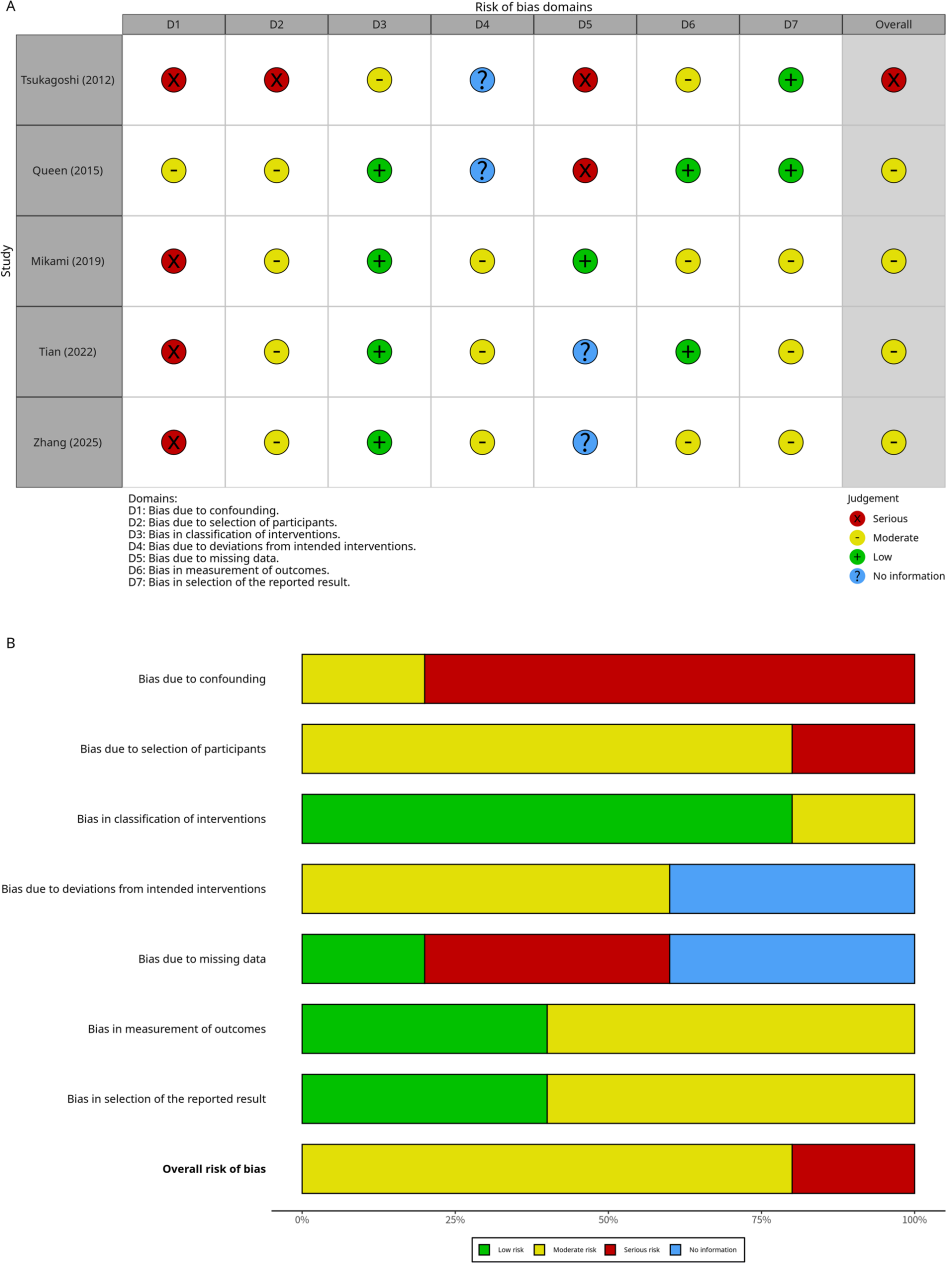
Risk of bias assessment using the ROBINS-I Tool for Non-Randomized Studies. **A** Traffic Light Plot of Domain-Level Risk-of-Bias Judgements. This panel displays the risk-of-bias judgements (low risk: green; moderate risk: yellow; serious risk: red; critical risk: purple) for each domain across individual included studies. **B** Summary Bar Plot of the Distribution of Risk-of-Bias Judgements. This panel shows the proportion (or weighted proportion) of studies judged as low, moderate, serious, or critical risk within each bias domain, including overall risk of bias

The risk of bias assessment for the 17 included randomized controlled trials, evaluated using the RoB 2 tool (Fig. 2), revealed that no study achieved an overall low risk of bias.Most trials (*n* = 14) raised some concerns overall, primarily driven by potential biases in the randomization process (D1) and measurement of the outcome (D4), while three studies were judged as high risk due to deviations from intended interventions and missing outcome data. In contrast, the five non-randomized studies assessed with the ROBINS-I tool (Fig. 3) showed predominantly moderate (*n* = 3) or serious (*n* = 2) overall risk of bias, with consistent concerns in confounding (D1) and selection of participants (D2), though biases in outcome measurement and reported results were generally low to moderate. Overall, the evidence base is limited by moderate to high risk of bias across both randomized and non-randomized designs. For hip range of motion, evidence certainty was very low (downgraded due to risk of bias and inconsistency). A summary of all pooled effect estimates is presented in Table 3.

**Table 3:** Summary of pooled effect estimates for all outcomes

Outcome	Subgroup/time point	No. of studies (participants)	Pooled effect (95% CI)	I²	Direction (favours exercise)
Hip abduction strength (SMD)	Short-term (< 3 months)	9 (287)	−0.78 (−1.19 to −0.36)	62.4%	Yes
	Mid-term (3–6 months)	4 (108)	−0.69 (−1.10 to −0.29)	0.0%	Yes
	Long-term (> 12 months)	3 (89)	−0.29 (−0.75 to 0.17)	1.2%	No (NS)
Hip flexion strength (SMD)	All follow-up	3 (113)	0.10 (−0.27 to 0.47)	0.0%	No
Harris Hip Score (MD)	1 week	1 study arm	−2.74 (−3.76 to −1.72)	–	Yes
	2 weeks	2 (78)	−4.62 (−5.70 to −3.55)	0.0%	Yes
	4 weeks	1 study arm	−7.67 (−8.97 to −6.37)	–	Yes
	6 weeks	1 study arm	−17.83 (−22.34 to −13.32)	–	Yes
	8 weeks	1 study arm	−2.22 (−6.82 to 2.38)	–	No (NS)
	12 weeks	2 (data not shown)	−3.05 (−5.38 to −0.72)	0.0%	Yes
	1 year	2 (72)	−17.94 (−49.13 to 13.25)	99.1%	No (NS)
Hip range of motion (MD)	4 weeks (random-effects)	2 (125)	−5.82 (−24.45 to 12.82)	94.4%	No (NS)

*SMD* Standardized mean difference (Hedges’ g), *MD* Mean difference, *CI* Confidence interval, *NS* Not significant (95% CI includes null). A negative effect estimate favours the structured exercise intervention over control

### Hip abduction strength

**Fig. 4:**
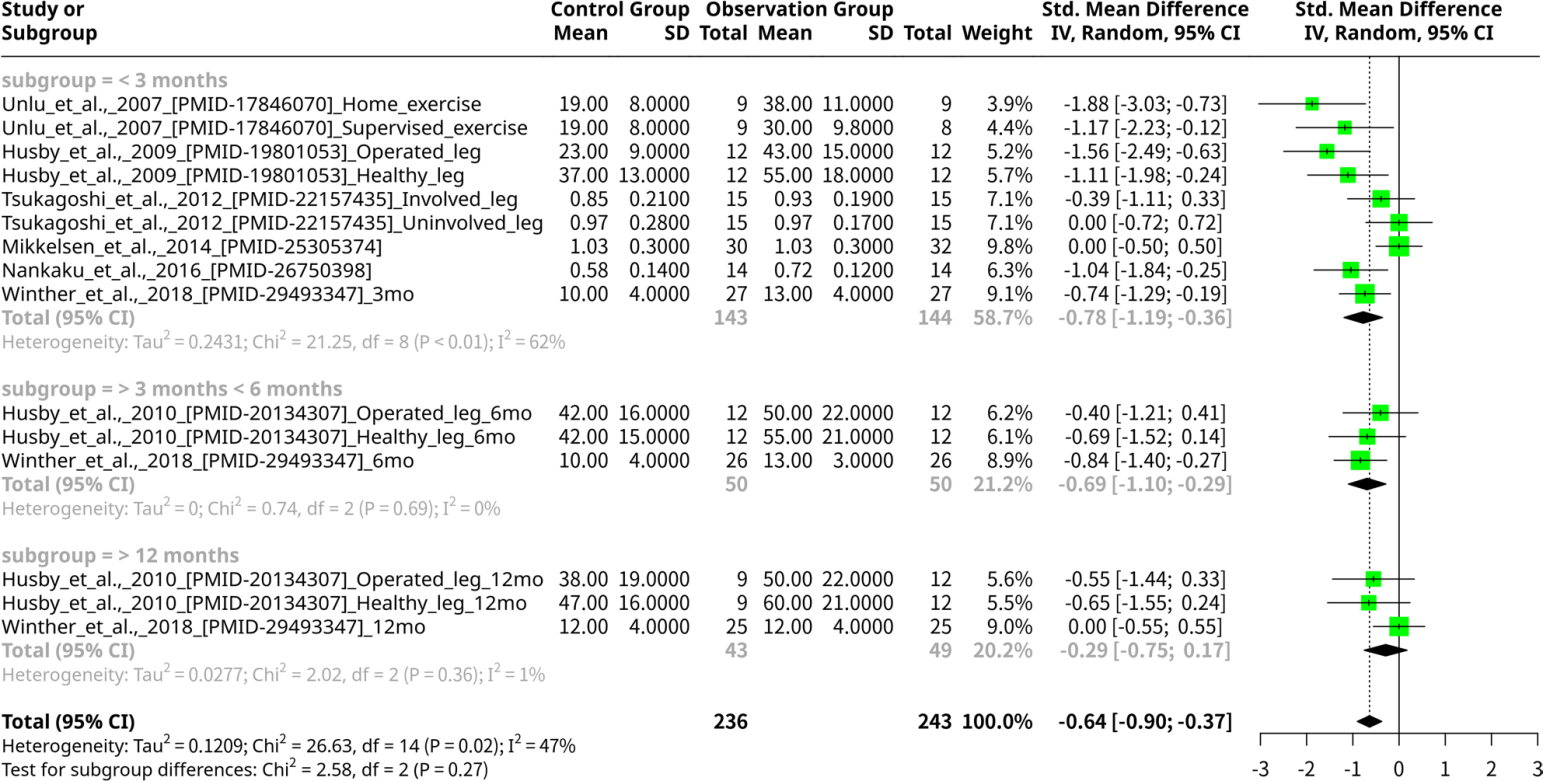
Forest plot of subgroup analysis for hip abduction strength by follow-up duration. Individual study estimates are shown as squares, and subgroup pooled estimates as diamonds (Hedges’ g with 95% confidence intervals). A negative SMD favours the structured exercise intervention over the control; accordingly, the left side of the null line represents the direction favouring the intervention. Subgroups are labelled with follow-up definitions: short-term (< 3 months), mid-term (3–6 months), and long-term (> 12 months), along with total participant numbers (n)

In the subgroup analysis stratified by follow-up duration, a random-effects model using Hedges’ g was applied. The analysis of studies with follow-up shorter than 3 months demonstrated a standardized mean difference (SMD) of −0.78 (95% CI: −1.19 to −0.36), indicating a significant effect in favor of the exercise intervention, with considerable heterogeneity (I² = 62.4%, τ² = 0.24). For the subgroup with follow-up between 3 and 6 months, the pooled SMD was − 0.69 (95% CI: −1.10 to −0.29), also showing a significant benefit with no observed heterogeneity (I² = 0.0%, τ² = 0.00). Conversely, in the long-term subgroup with follow-up exceeding 12 months, the effect size diminished to a non-significant SMD of −0.29 (95% CI: −0.75 to 0.17), with negligible heterogeneity (I² = 1.2%, τ² = 0.03). The test for differences between these subgroups was not statistically significant (Q = 2.58, *p* = 0.27) Based on the ICEMAN tool, the credibility of the subgroup analysis by follow-up duration for hip abduction strength was rated as low due to the absence of a pre-specified direction of effect modification, lack of prior evidence supporting time-dependent effects, and the non-significant test for subgroup differences (*p* = 0. 27) [PMID-32213509].The distribution of effect sizes across these time points is visually summarized in the forest plot (Fig. 4). A negative SMD favours the exercise intervention group over the control).

**Fig. 5:**
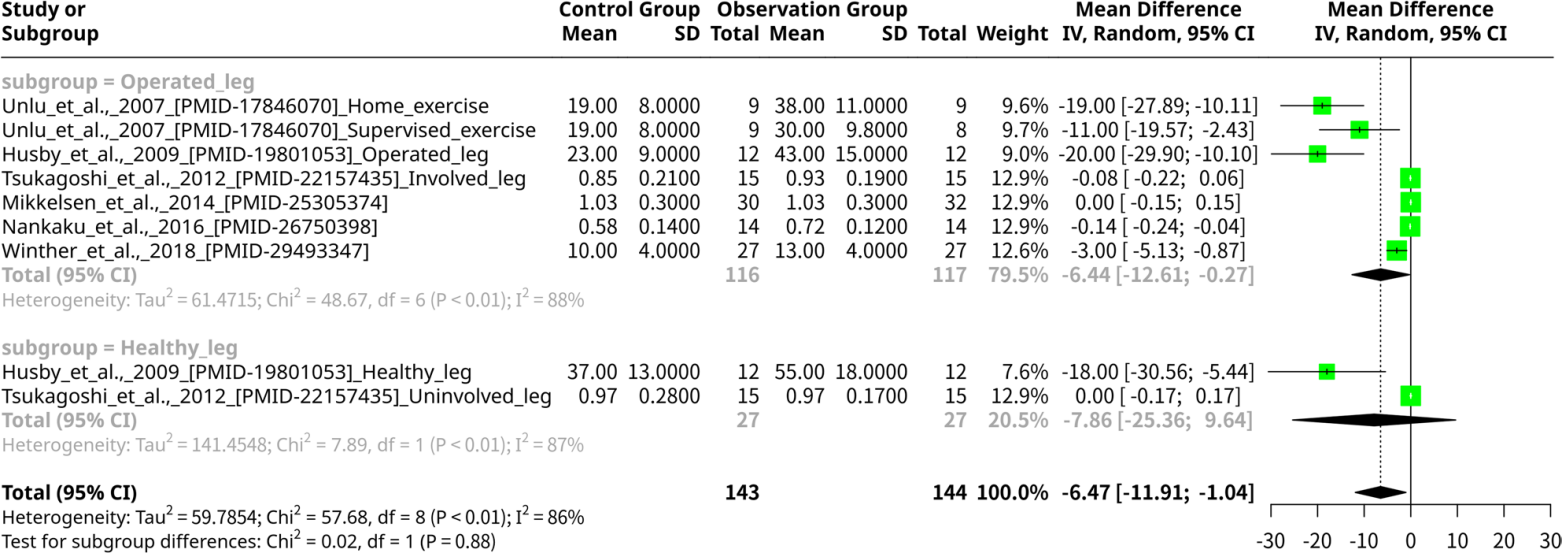
Forest Plot of Subgroup Analysis for Hip Abduction Strength by Limb Status in Studies with ≤ 3 Months Follow-up. The plot displays individual study estimates and subgroup pooled estimates (Hedges’ g) for the operated limb and the healthy (non-operated) limb. A negative SMD favors the exercise intervention. Each subgroup is labeled with the assessed limb (operated vs. healthy) and total number of participants (n)

For studies with a follow-up duration of ≤ 3 months, a subgroup analysis was performed based on the assessed limb (operated vs. healthy). The pooled analysis of 9 datasets (287 observations) using a random-effects model yielded a significant overall standardized mean difference (SMD) of −0.78 (95% CI: −1.19 to −0.36; *p* = 0.0003), with substantial heterogeneity (I² = 62.4%, τ² = 0.24). In the subgroup of the operated limb (k = 7), the pooled SMD was − 0.86 (95% CI: −1.34 to −0.37), indicating a significant effect favoring the intervention group. The healthy limb subgroup (k = 2) showed a non-significant pooled SMD of −0.52 (95% CI: −1.61 to 0.56). The test for subgroup differences between the operated and healthy limbs was not statistically significant (Q = 0.30, *p* = 0.58). Based on the ICEMAN tool, the credibility of the subgroup analysis by assessed limb for hip abduction strength was rated as very low because the analysis was not pre-specified, only two studies contributed to the healthy limb subgroup, and the test for subgroup differences was non-significant (*p* = 0. 58) [PMID-32213509]. The distribution of effect sizes for this analysis is presented in Fig. 5.

### Hip flexion strength

**Fig. 6:**
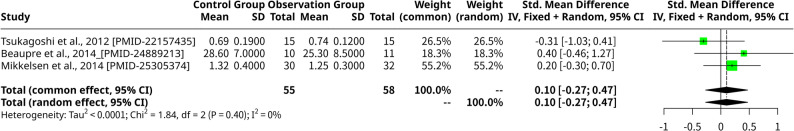
Forest Plot of Meta-Analysis for Hip Flexion Strength. The plot displays the standardized mean difference (Hedges’ g) with 95% confidence intervals for individual studies and the pooled estimate from a random-effects model. A positive SMD favors the exercise intervention. The overall sample size and follow-up range (preoperative to 10 weeks) are indicated in the plot

Only the random-effects model is reported, as the common-effect model yields an identical result due to negligible heterogeneity (τ² = 0. 00). The meta-analysis of hip flexion strength, comprising data from 3 studies involving 113 participants, was conducted. The pooled SMD calculated using Hedges’ g was 0.10 (95% CI: −0.27 to 0.47), indicating no statistically significant difference between the intervention and control groups (z = 0.54, *p* = 0.59). Heterogeneity among the studies was negligible, with an I² statistic of 0.0% (Q = 1.84, *p* = 0.40). The distribution of individual and pooled effect sizes is presented in the forest plot (Fig. 6).

### Harris hip score

**Fig. 7:**
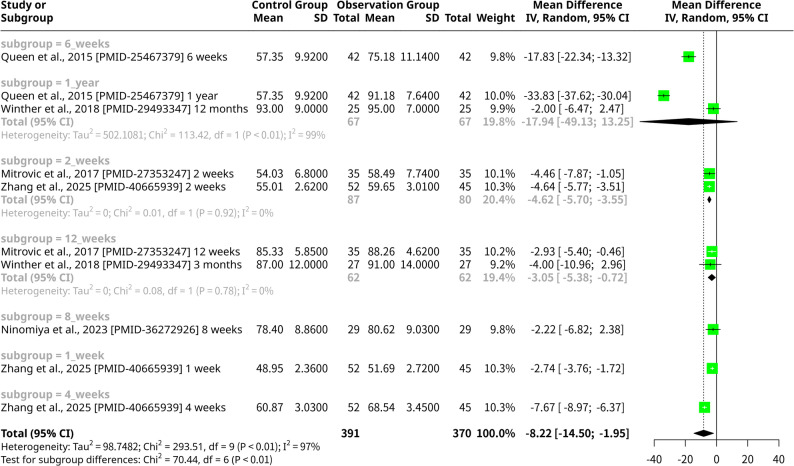
Forest plot of mean differences in Harris Hip Score stratified by follow-up time. Pooled mean differences and 95% confidence intervals are shown across seven postoperative follow-up time points. A negative mean difference (MD) favours the structured exercise intervention over the control; thus, the left side of the null line indicates the direction favouring the intervention. Each subgroup is labelled with its follow-up time point (e.g., 1 week, 2 weeks) and total participant number (n)

The meta-analysis of ten datasets demonstrated considerable variation in treatment effects on the Harris Hip Score across different postoperative follow-up periods (Fig. 7). At the one-week follow-up (1 study arm), the mean difference (MD) was − 2.74 (95% CI: −3.76 to −1.72). The two-week follow-up subgroup (2 study arms) showed an MD of −4.62 (95% CI: −5.70 to −3.55, I² = 0.0%). For the four-week follow-up (1 study arm), the MD was − 7.67 (95% CI: −8.97 to −6.37. At the 6–8 weeks follow-up subgroup (2 study arms), the MDs were − 17. 83 (95% CI: −22. 34 to −13. 32) and − 2. 22 (95% CI: −6. 82 to 2. 38), respectively. these two study arms, originally reported at 6 weeks and 8 weeks, are now combined into the 6–8 weeks follow-up subgroup to reduce unnecessary stratification. The twelve-week follow-up subgroup (2 study arms) had an MD of −3.05 (95% CI: −5.38 to −0.72, I² = 0.0%). The one-year follow-up subgroup (2 study arms) demonstrated the largest point estimate, with an MD of −17.94, although with a wide confidence interval (95% CI: −49.13 to 13.25) and considerable heterogeneity (I² = 99.1%). The test for subgroup differences was statistically significant (*p* < 0.0001).

### Hip joint range of motio

**Fig. 8:**
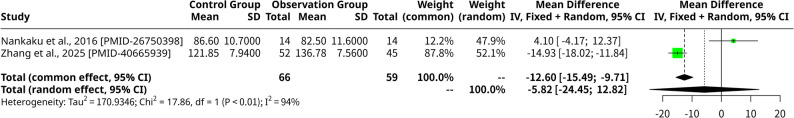
Forest plot of the meta-analysis for hip joint range of motion using a random‑effects model. Individual and pooled mean differences (MD) with 95% confidence intervals are shown from two studies. A negative MD favours the structured exercise intervention; therefore, the left side of the null line represents the direction favouring the intervention. Each study is labelled with its follow-up time point (4 weeks) and sample size (n). The substantial heterogeneity is visually evident from the non‑overlapping confidence intervals of the individual study estimates

The meta-analysis of two studies (k = 2, *n* = 125 participants) investigating the effect of postoperative rehabilitation on hip joint range of motion was conducted using a random-effects model (Fig. 8). Random effects model, which accounts for between-study heterogeneity, showed a non-significant pooled MD of −5. 82 (95% CI: −24. 45 to 12. 82, *p* = 0. 5406). Statistically significant heterogeneity was observed between the two included studies (Q = 17.86, *p* < 0.0001; I² = 94.4%, tau² = 170.93).

### Other clinical indicators

Secondary outcomes (e.g., WOMAC, 6MWT, stair climbing, TUG, quality of life, pain) generally improved with structured exercise, particularly in the short- to medium-term, with high adherence and favorable safety [7–29].

## Discussion

This systematic review and meta-analysis demonstrated that structured postoperative exercise significantly improves hip abduction strength in the short- and mid‑term (SMD ‑0.78 and ‑0.69, respectively) and enhances Harris Hip Score at early time points (e.g., 2, 4, 6, 12 weeks), but does not significantly affect hip flexion strength (SMD 0.10, 95% CI: ‑0.27 to 0.47). Long‑term effects on strength and function remain uncertain. We acknowledge that the intervention category ‘structured postoperative exercise’ encompasses highly heterogeneous modalities (progressive resistance training, walking programs, balance training, multimodal rehabilitation, exercise plus nutrition, treadmill/electrical stimulation), which may reduce clinical interpretability when pooled.

Our findings differ notably from those of Saueressig et al., 2021, but these discrepancies reflect advancements in the evidence base and more granular analytical approaches rather than contradiction. The divergence stems from an enlarged evidence base including newer studies (e.g., Nankaku et al., 2016; Winther et al., 2018; Tsukagoshi et al., 2012) and our time‑stratified, limb‑specific analysis. We treated multiple time points from the same study (e.g., Husby et al., 2009; Winther et al., 2018) as independent data points within respective temporal subgroups, adhering to the principle that effects at 6 weeks, 6 months, and 12 months are clinically distinct outcomes. In contrast, the prior review selected a single time point “closest to” a predefined milestone, which may blend effects from different recovery phases. We acknowledge that treating repeated observations as independent may artificially narrow confidence intervals—a limitation. We did not apply multilevel models or meta‑regression because each data point came from distinct participant groups and the number of studies per subgroup was insufficient.

Our time‑ and limb‑stratified analysis revealed patterns obscured in broader syntheses. While Saueressig et al. presented time‑stratified results, we separated analyses for operated vs. healthy limb within the early (≤ 3 months) period, revealing a strong effect on the operated limb (SMD ‑0.86) that was diluted in prior analyses. Our stricter, non‑overlapping follow‑up thresholds (≤ 3 months, 3‑6 months, > 12 months) provide clearer insight into recovery trajectory: a pronounced early effect that diminishes long‑term. The prior review’s categories (e.g., “Closest to 26 wk”) are more heterogeneous, contributing to wider confidence intervals. Differences also stem from intervention protocols; our included studies used maximal strength training (Husby, Winther) or specific hip external rotator programs (Nankaku), representing more intensive approaches. Measurement heterogeneity exists (isometric torque vs. dynamic strength), standardized via SMD. Despite numerical differences, both analyses converge on a time‑dependent effect, with larger benefits near the intervention period and attenuation at one year. Our analysis provides stronger, more precise evidence for short‑term benefit (SMD ‑0.78, CI: ‑1.19 to ‑0.36).

For hip flexion strength, our pooled effect (SMD 0.10, 95% CI: ‑0.27 to 0.47) aligns with Saueressig et al. (2021) (SMD ‑0.20, 95% CI: ‑1.29 to 0.90); both CIs encompass zero. Our analysis incorporated different studies (Tsukagoshi et al., 2012; Beaupre et al., 2014) focused on earlier time points, yielding a narrower CI. The prior analysis included Beck et al. (2019) and Johnsson et al. (1988) at later follow‑up, producing a wide CI and greater uncertainty. When interpreting heterogeneity for hip flexion strength, an I² of 0% does not automatically imply homogeneity of effect directions: the three studies had point estimates on opposite sides of the null (two positive, one negative) but wide overlapping CIs, reflecting limited precision rather than true consistency. We therefore complemented I² with qualitative examination of the forest plot.

Comparing temporal patterns between HHS and hip abduction strength reveals convergent and divergent trends. In the short‑to‑medium term (up to 6 months), both outcomes improved significantly. The notable HHS peak at 6 weeks (MD ‑17.83) may represent a critical early phase where gains in pain relief, mobility, and function—underpinned by recovering muscle strength—are most pronounced. However, long‑term trajectories differ: hip abduction strength becomes non‑significant at > 12 months (SMD ‑0.29), suggesting attenuation as controls catch up. For HHS at one year, the point estimate remained large (MD ‑17.94) but with an extremely wide CI and profound heterogeneity (I² = 99.1%), indicating extreme inconsistency rather than robust benefit. Heterogeneity further distinguishes these outcomes: HHS exhibited extreme heterogeneity at 1 year, with a significant subgroup difference (*p* < 0.0001), confirming follow‑up time as a major moderator. Hip abduction strength showed moderate short‑term and negligible longer‑term heterogeneity, with non‑significant subgroup difference (*p* = 0.27).

Hip ROM analysis is inconclusive and dominated by extreme heterogeneity (I²=94.4%). The conflicting results between common‑effect (MD ‑12.60) and random‑effects (MD ‑5.82, NS) models directly stem from this heterogeneity. One study measured single‑plane hip flexion angle, the other a composite ROM score; interventions also differed (external rotator exercise vs. stepped rehabilitation). This lack of standardization makes synthesis problematic. No reliable ROM effect corroborates the early benefits seen in strength and HHS, and long‑term ROM data are absent. Thus, current evidence on active hip ROM is too fragmented to support any definitive conclusion.

Secondary outcomes (WOMAC, gait speed, 6MWT, stair climbing, TUG, quality of life, pain) generally improved with structured exercise, particularly short‑ to medium‑term, with high adherence and favourable safety [7–29]. Significant improvements in WOMAC up to 24 weeks, gains in gait speed, 6MWT distance, stair climbing efficiency, and TUG performance collectively mirror the pattern of early functional recovery captured by HHS up to 12 weeks [7–16, 18, 21–24, 28, 29]. Meaningful improvements in physical and overall quality of life [20, 25, 27] and pain reduction [25] further substantiate patient‑perceived value.

Bilateral improvements in knee extensor strength, hip extension torque, and rate of force development [11, 19] contrast with non‑significant hip flexion strength, suggesting that exercise efficacy is muscle‑group‑specific. “Sustained” endurance and strength gains over months [14, 19] somewhat contrast with diminishing abduction strength beyond 12 months; however, long‑term evidence for other indicators is not extensively documented. High adherence and favourable safety (e.g., reduced thrombosis, falls) [12, 25] address feasibility and harm.

## Limitations

Overall evidence quality is limited by moderate‑to‑high risk of bias. According to GRADE, short‑ and mid‑term hip abduction strength benefits are moderate certainty (downgraded for risk of bias); long‑term benefit is low certainty (imprecision + risk of bias). Hip flexion strength evidence is low certainty (imprecision, small number of studies). HHS improvements up to 12 weeks are moderate certainty; one‑year evidence is very low certainty (extreme heterogeneity, imprecision, risk of bias). Limitations include: moderate‑to‑high risk of bias across studies (RoB 2 and ROBINS‑I); small numbers for hip flexion (k = 3, *n* = 113) and ROM (k = 2, *n* = 125); substantial heterogeneity in exercise protocols, parameters, and outcome measurements; sparse long‑term follow‑up with extreme heterogeneity (especially HHS at 1 year); publication bias not formally assessed due to < 10 studies per analysis. These ratings highlight the need for future high‑quality trials with standardised protocols and longer follow‑up to confirm short‑term benefits and clarify long‑term effects.

## Conclusions

Structured postoperative exercise provides clinically meaningful short‑ to mid‑term improvements in hip abduction strength and early patient‑reported function, but its long‑term efficacy and impact on hip flexion strength remain unconfirmed.

## Data Availability

The dataset generated and analysed during the current study is available from the corresponding author on reasonable request.

## Abbreviations

THA: Total Hip Arthroplasty
HHS: Harris Hip Score
SMD: Standardized Mean Difference
MD: Mean Difference
CI: Confidence Interval
ROM: Range of Motion
RCT: Randomized Controlled Trial
REML: Restricted Maximum-Likelihood
6MWT: 6-Minute Walk Test
WOMAC: Western Ontario and McMaster Universities Osteoarthritis Index
HOOS: Hip disability and Osteoarthritis Outcome Score
EQ-5D: EuroQol-5 Dimensions
SF-36: 36-Item Short Form Survey
TUG: Timed Up and Go Test
VAS: Visual Analog Scale
JHEQ: Japanese Orthopaedic Association Hip Disease Evaluation Questionnaire
PRISMA: Preferred Reporting Items for Systematic Reviews and Meta-Analyses
PICOS: Population, Intervention, Comparison, Outcomes, Study Design
BMI: Body Mass Index
